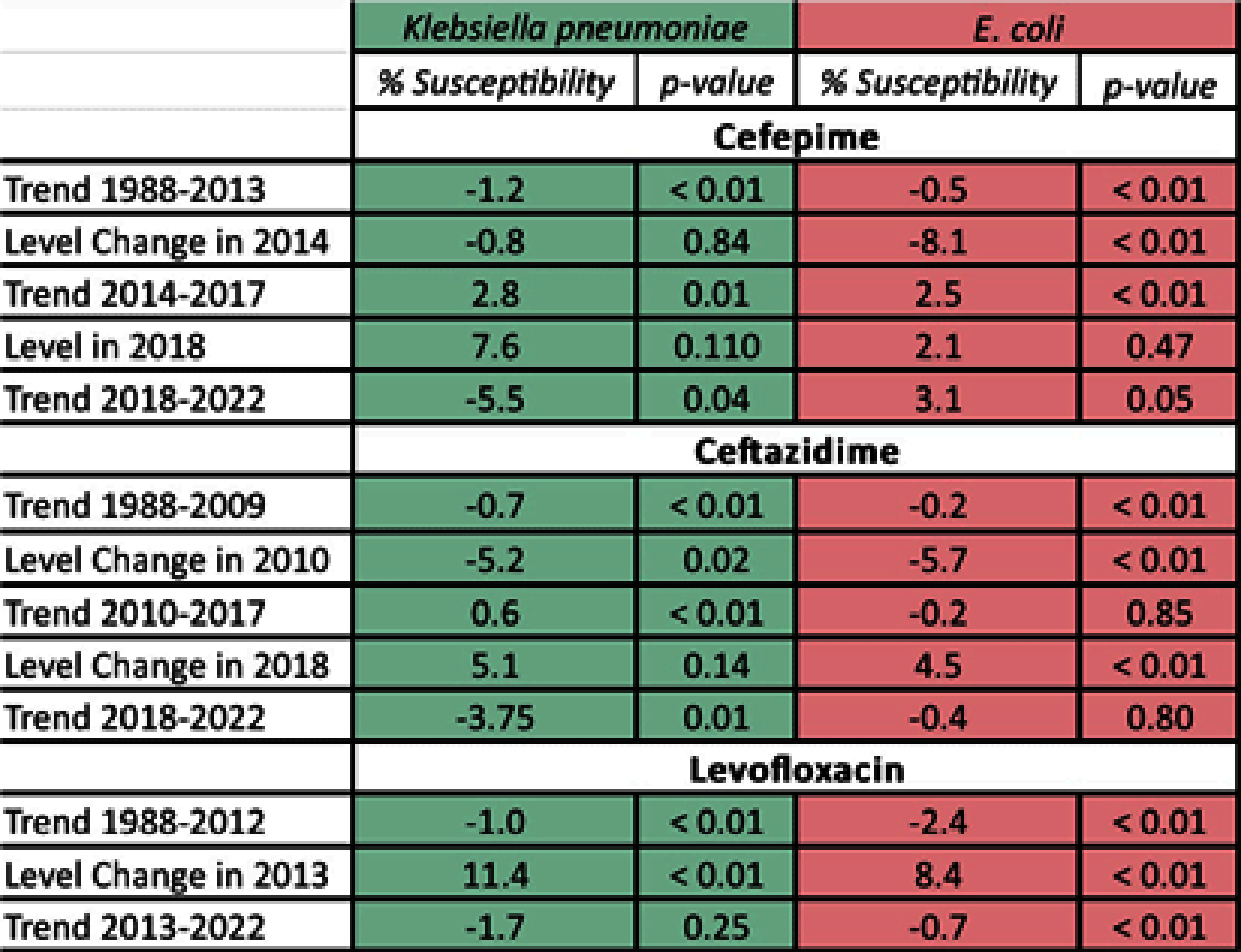# Impact of MIC Breakpoint Changes for Enterobacterales on Trends of Antibiotic Susceptibilities in An Academic Medical Center

**DOI:** 10.1017/ash.2024.171

**Published:** 2024-09-16

**Authors:** David Evans, Marisa Winkler, Eileen Burd, Ashley Jones, Trinh Vu, James Steinberg, Jesse Jacob

**Affiliations:** Emory University, Rollins School of Public Health; Department of Pathology and Laboratory Medicine, Emory University School of Medicine, Atlanta GA; Emory University Hospital Midtown; Emory University

## Abstract

**Background:** The Clinical & Laboratory Standards Institute (CLSI) recommends use of annual antibiograms to help guide empiric antibiotic therapy. Because CLSI periodically updates minimum inhibitory concentration (MIC) breakpoints, we assessed the impact of these updates on longitudinal trends in antibiotic susceptibility rates for Escherichia coli and Klebsiella pneumoniae at a single academic medical center in Atlanta, GA. **Methods:** Susceptibilities for cefepime, ceftazidime, and levofloxacin in E. coli and K. pneumoniae were extracted from hospital antibiograms from 1988 to 2022. Starting in 1995, intensive care units (ICUs) and wards had separate annual antibiograms, which we combined using weighted averages to create annual overall hospital antibiograms. After summarizing the frequency of isolates tested and susceptibilities using medians and interquartile ranges (IQR), we conducted an interrupted time series analysis using linear segmented regression models, to evaluate the level changes and trends in susceptibility, before and after CLSI MIC breakpoints were updated for ceftazidime (2010 and 2017), cefepime (2014 and 2017), and levofloxacin (2013). **Results:** Among 21,214 E. coli, there was a median of 291 [IQR: 104, 555] isolates tested annually. Similarly, among 8,686 K. pneumoniae isolates, the median was 125 per year (IQR: 76, 178). Prior to the MIC breakpoint changes, baseline susceptibility trends of both organisms to all 3 antibiotics significantly declined at a rate between 0.2% to 2.4% per year (Table 1). For cefepime (Figure 1), susceptibility decreased annually during 1988 – 2013 for both E. coli (-0.5%) and K. pneumoniae (-1.2%). There were no significant level changes but there were trend changes after 2018, for E. coli (+2.1%) and K. pneumoniae (– 5.5%). For ceftazidime (Figure 2), significant level changes occurred after 2010 for both organisms (E. coli: -5.7%; K. pneumoniae: -5.2%). For levofloxacin (Figure 3), the breakpoint update in 2013 lead to significant level change in susceptibility (E. coli: +8.4%; K. pneumoniae: +11.4%). **Conclusion:** Overall, we observed a consistent decrease in antibiotic susceptibility in E. coli and K. pneumoniae over three decades, with immediate increases in the level change of susceptibility when MIC breakpoints were changed, followed by a decreasing trend. These findings highlight the importance of longitudinal surveillance and MIC breakpoint changes to inform antimicrobial stewardship strategies.

**Figure:**
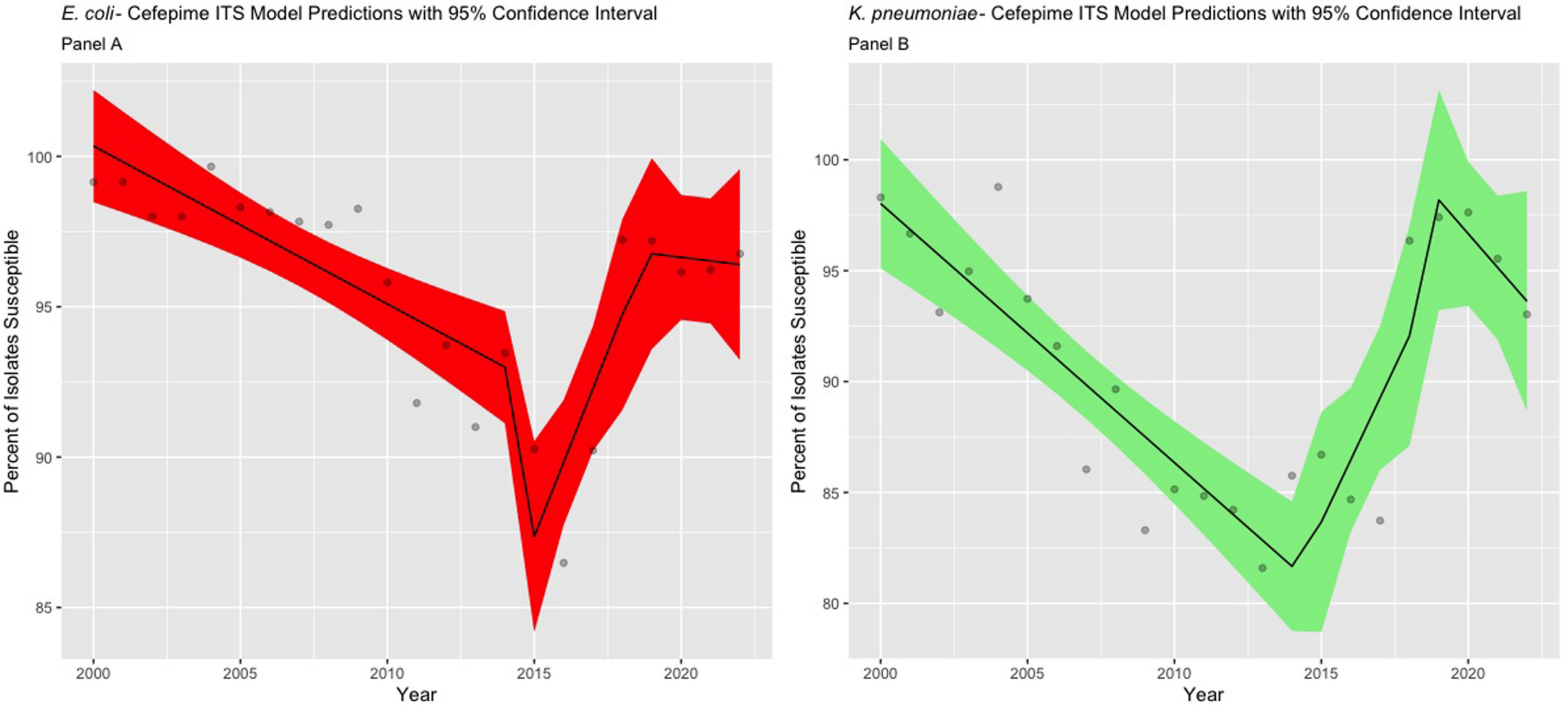

**Figure:**
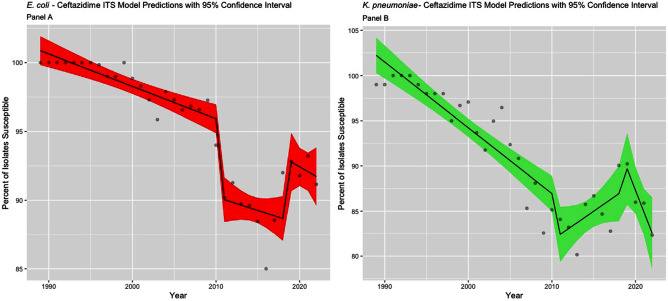

**Figure:**
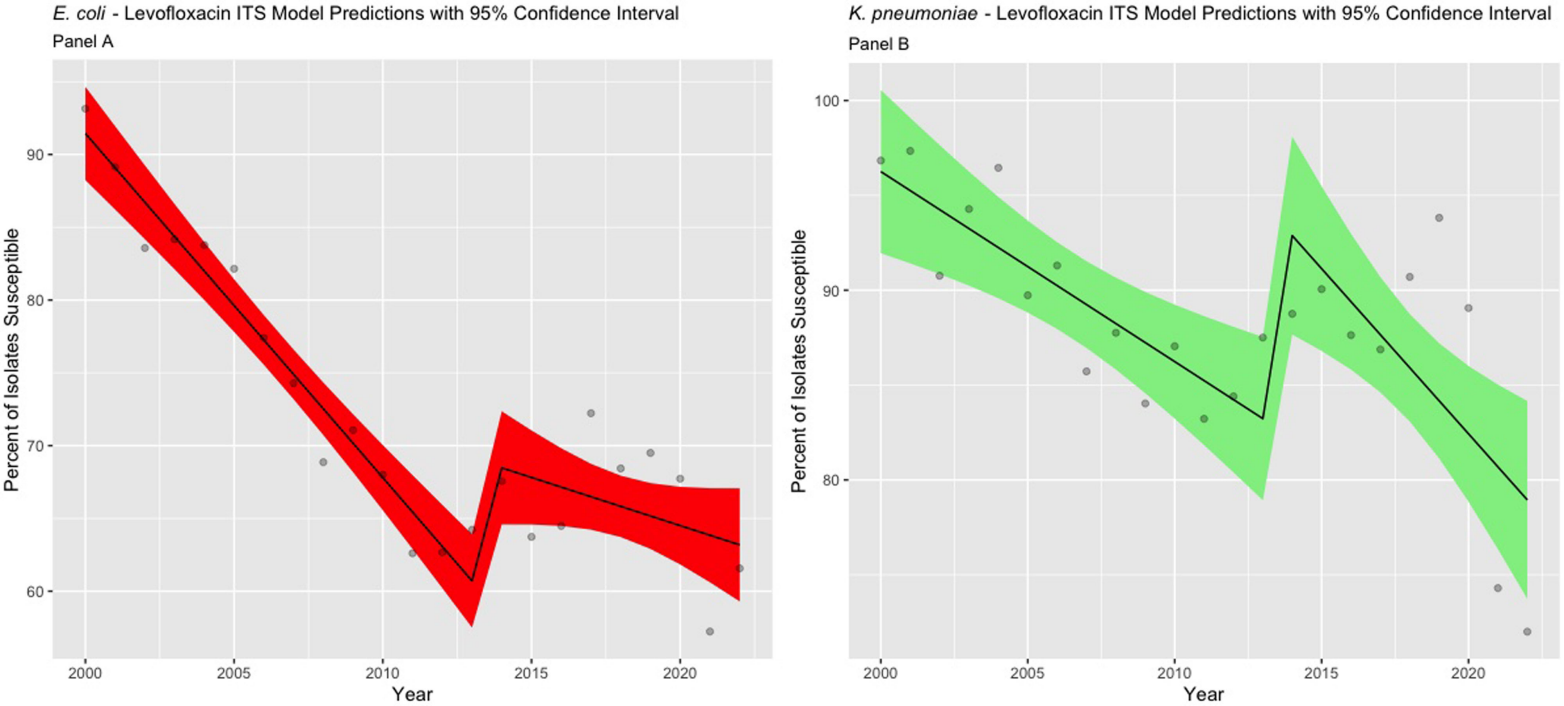

**Figure:**